# Case report: Recurrent severe mitral regurgitation due to ruptured artificial chords after transapical Neochord mitral valve repair

**DOI:** 10.3389/fcvm.2022.985644

**Published:** 2022-11-09

**Authors:** Mi Zhou, Ka-chun Un, Chun Ka Wong, On Yat Wong, David Chung Wah Siu, Lixue Yin, Daniel Tai-leung Chan, Simon Cheung Chi Lam

**Affiliations:** ^1^Cardiology Division, Department of Medicine, Queen Mary Hospital, The University of Hong Kong, Hong Kong, Hong Kong SAR, China; ^2^Department of Anesthesiology, Queen Mary Hospital, The University of Hong Kong, Hong Kong, Hong Kong SAR, China; ^3^Cardiovascular Ultrasound and Non-Invasive Cardiology Department, Sichuan Provincial People's Hospital, Chengdu, China; ^4^Department of Cardiothoracic Surgery, Queen Mary Hospital, The University of Hong Kong, Hong Kong, Hong Kong SAR, China

**Keywords:** valvular heart disease, mitral regurgitation, mitral valve (MV) repair, cardiac intervention, echocardiography

## Abstract

Transapical Neochord mitral valve repair has been proven to be a technically safe procedure to correct primary mitral regurgitation (MR). Recurrent MR due to ruptured artificial chords is rare. Here, we present 2 cases of recurrent severe MR due to the detached or partially ruptured artificial chords after the Neochord procedure.

## Introduction

The early phase of degenerative mitral regurgitation (MR) is characterized by compensatory adaptions, demonstrated with normal left ventricular ejection fraction (LVEF), dilated left ventricle, or eccentric hypertrophy (1). In this stage, most of the patients are asymptomatic or present with non-specific symptoms related to lung infections (cough, sputum, fever). Gradually, an uncorrected MR would lead to irreversible remodeling of the left ventricle and present with cardiac dysfunction, pulmonary hypertension, and poor outcome (1, 2). To date, the evidence of transapical Neochord (DS1000 System) mitral valve repair in reducing MR in suitable patients with chronic severe primary MR has been well-established (3, 4). Further, several clinical trials have been carried out in European and United States to compare the safety and performance of the NeoChord procedure with conventional open surgical repair (ClinicalTrials.gov NCT01777815, NCT02803957). Here, we report two patients with recurrent severe MR after the Neochord procedure due to the ruptured or loosed artificial chords.

### Patient 1: Recurrent severe MR due to detached artificial chords

**Figure 1:**
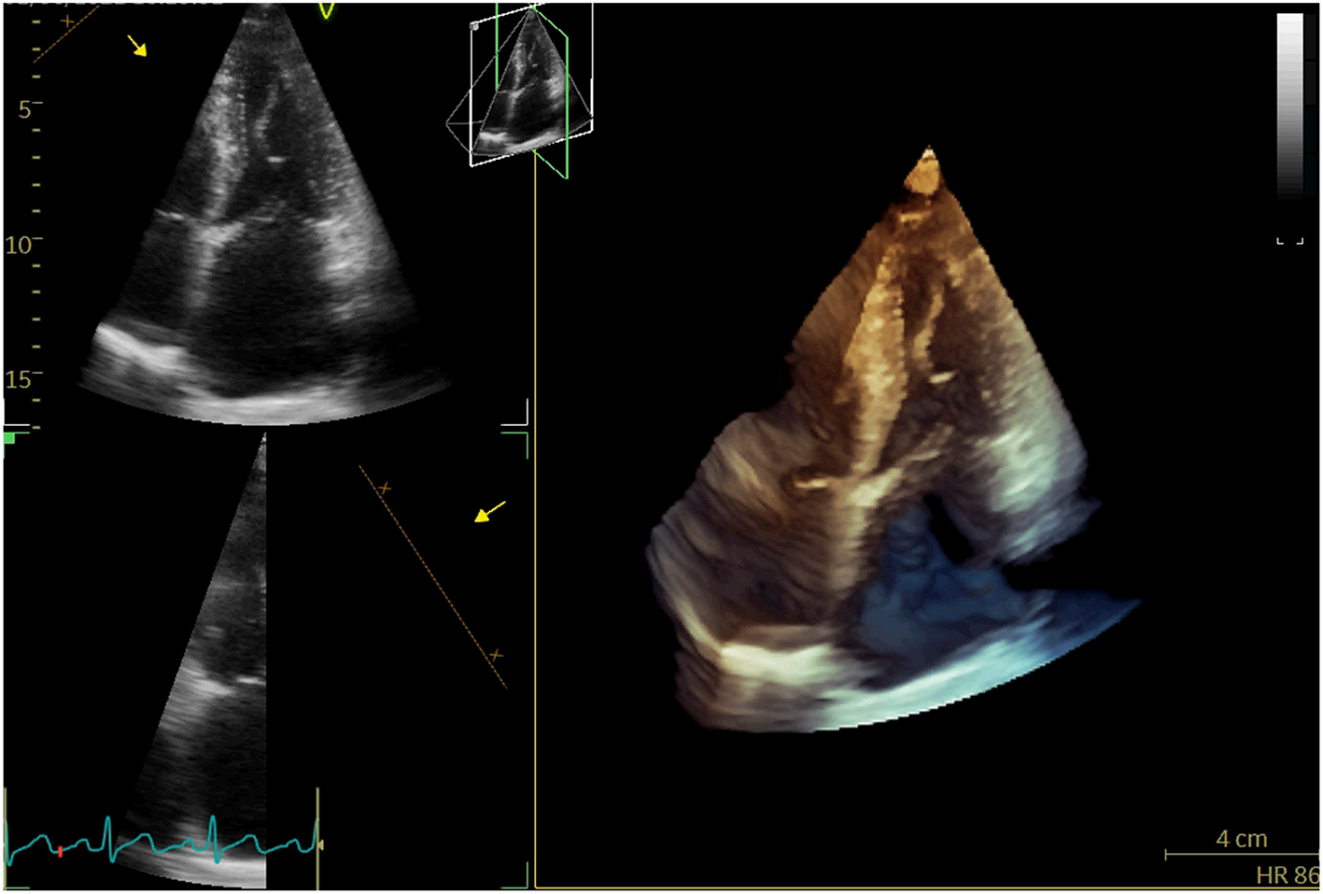
3D-TTE revealed detached artificial chords.

A 71-year-old woman repeatedly presented to the outpatient department for cough, whitish sputum, and progressive shortness of breath (SOB) since 2018. Physical examination indicated that vital signs were stable (BP: 124/71 mmHg, Temperature: 37.0°C, *P*: 70 beats/min, SPO_2_: 98%), and lower limb edema. ECG: normal. Cardiac enzyme: unremarkable. Chest X-ray: bilateral lower zone haziness, blunted bilateral costophrenic angle. The symptoms were resolved after treatment with augmentin, Lasix, and oxygen treatment (2L/min). Subsequent transthoracic echocardiography (TTE) and transesophageal echocardiography (TEE) showed that normal-sized left ventricle with preserved LVEF (55–60%); severe anterior directed MR due to prolapse P2 and P3 segment. She underwent Neochord repair with 4 artificial chords implanted, residual trivial MR with a mean trans-mitral gradient of 4 mmHg. The patient followed a regular postoperative valvular evaluation by TTE every 6 months after being discharged from the hospital. Twenty-four months after discharge, she was admitted to the cardiac clinic again for a severe SOB and heart failure symptoms (New York heart association functional class 3–4) and decreased exercise tolerance. Conventional and 3-D TTE showed severe anterior directed MR due to the detached artificial chords (Figure 1).

### Patient 2: Recurrent severe MR due to partially ruptured artificial chords

**Figure 2:**
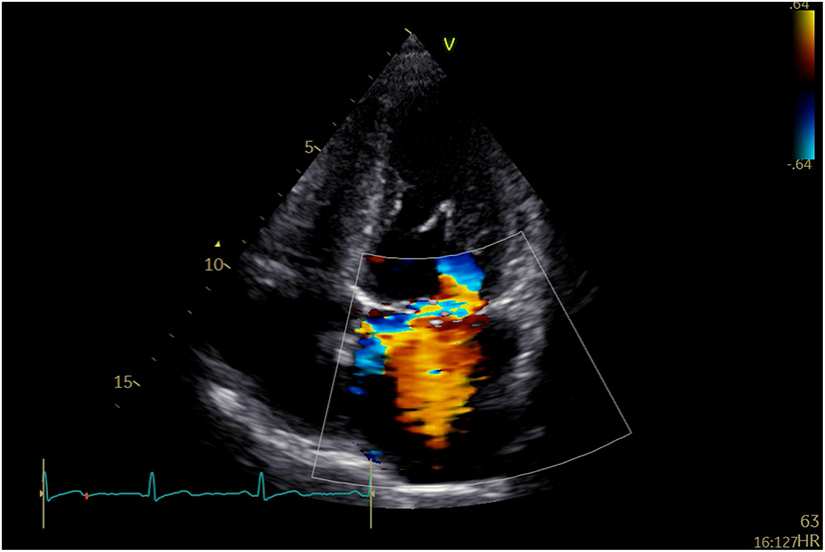
TTE revealed recurrent severe MR due to partially ruptured artificial chords.

A 58-year-old man was repeatedly referred to the respiratory clinic for cough, sputum, and fever since 2003. He was founded with posterior mitral valve prolapse with mild MR in 2013. He felt palpitation and exertional dyspnea in March 2019. TTE and TEE revealed severe MR due to P1, and P2 leaflet prolapse; borderline LV cavity size with LVEF ~65%. Holter: sinus tachycardia, several episodes of supraventricular contractions, and premature ventricular contractions. On examination, his vital signs were stable (BP: 129/71 mmHg, *p*: 86 beats/min). He underwent Neochord mitral valve repair with 3 artificial chords, with residual trivial MR and a mean trans-mitral gradient of 2–4 mmHg. Thirty months after he was discharged from the hospital, he was admitted to the hospital again for an exertional SOB. TTE indicated severe anterior directed MR due to partially ruptured artificial chords (Figure 2).

## Discussion

Off-pump transapical Neochord mitral valve repair has been proven to be a technically safe and achievable procedure to correct MR from grade 3+/4+ to ≤ 2+ (5). Type A or type B anatomy which featured isolated-/multi-segments prolapse or flail of the posterior mitral leaflet was considered the optimized selection for Neochord implantation. Type C and type D anatomy was defined as anterior or bileaflet prolapse with or without paracommissural involvement and annular calcifications, which has been reported to meet 80% of the primary endpoint (moderate or less MR without the need for reoperation after Neochord repair at 1 year) compared to the type A (92%) and type B (88%) anatomy (4). In addition, the leaflet-to-annulus index (LAI) >1.25 will be taken into account before the procedure, as it is an essential predictor of a good outcome. Functional MR or MR due to endocarditis and ischemic heart disease should be excluded from Neochord mitral valve repair (3, 6). In our study, these two patients (type B MV anatomy and LAI ≥1.25) are preferred to receive the transapical Neochord repair compared to the conventional open mitral valve surgery because of its mini-invasive and off-pump nature. Procedural success was achieved in those two patients because residual MR evaluated immediately after surgery was trivial.

The incidence of recurrent severe MR after transapical Neochord procedure is 11% in the first year (4). Reasons for recurrent MR with a moderate or above degree are complicated, including inappropriate patient, new prolapse/re-prolapse of leaflet, leaflet tethering caused by LV dysfunction, calcification, untreated/residual leaflet prolapse, leaflet curling, over-tensioning of the treated leaflet, and ventricular dilation (7).

Recurrent MR due to ruptured artificial chords is rare. Early-intermediate rupture is defined as the rupture occurring within 1–3 years after the Neochord procedure. Reasons for early-intermediate rupture are unclear, probably due to the inflammatory response, chords injured intraoperatively, or intracardiac stress (8, 9). Late rupture is defined as rupture occurring 6–14 years postoperatively that may result from artificial chord calcification (10). Our two patients reached the primary outcome at 1 year and severe MR reoccurred at postoperative 24–30 months. Elective reoperation is scheduled for our two patients. In the absence of histopathological analysis of recurrent severe MR, we hypothesized that the early-intermediate failure was likely due to LV reverse remodeling leading to the loose of artificial chords or changes of intracardiac stress exceeding the strength of the artificial chords.

Although the results of transapical Neochord implantation for the correction of mitral regurgitation are very encouraging, more reliable evidence based on a large cohort study is needed to evaluate the short-term and long-term outcomes. In this context, our case series might provide a clue that artificial chords can loosen or partially rupture in the early- intermediate period after mitral valve repair.

## Data availability statement

The original contributions presented in the study are included in the article/supplementary material, further inquiries can be directed to the corresponding authors.

## Author contributions

MZ, DS, and LY contributed to the conception of the manuscript. MZ, CW, K-cU, OW, SL, and DC organized the data collection. MZ wrote the first draft of the manuscript. All authors contributed to manuscript revision, read, and approved the submitted version.

## Conflict of interest

The authors declare that the research was conducted in the absence of any commercial or financial relationships that could be construed as a potential conflict of interest.

## Publisher's note

All claims expressed in this article are solely those of the authors and do not necessarily represent those of their affiliated organizations, or those of the publisher, the editors and the reviewers. Any product that may be evaluated in this article, or claim that may be made by its manufacturer, is not guaranteed or endorsed by the publisher.
